# Synergistic biocementation: harnessing *Comamonas* and *Bacillus* ureolytic bacteria for enhanced sand stabilization

**DOI:** 10.1007/s11274-024-04038-3

**Published:** 2024-06-03

**Authors:** Adharsh Rajasekar, Cailin Zhao, Suowei Wu, Raphinos Tackmore Murava, Stephen Wilkinson

**Affiliations:** 1 Jiangsu Key Laboratory of Atmospheric Environment Monitoring and Pollution Control (AEMPC), Collaborative Innovation Center of Atmospheric Environment and Equipment Technology (CIC-AEET), Nanjing University of Information Science &Technology, Nanjing, 210044 China; 2 School of Geography and Environmental Sciences, University of Reading, Reading, RG6 6AH UK; 3 Faculty of Engineering and Information Sciences, University of Wollongong in Dubai, Dubai, UAE

**Keywords:** MICP, Biocementation, Bacterial synergy, Calcium carbonate, Urease

## Abstract

Biocementation, driven by ureolytic bacteria and their biochemical activities, has evolved as a powerful technology for soil stabilization, crack repair, and bioremediation. Ureolytic bacteria play a crucial role in calcium carbonate precipitation through their enzymatic activity, hydrolyzing urea to produce carbonate ions and elevate pH, thus creating favorable conditions for the precipitation of calcium carbonate. While extensive research has explored the ability of ureolytic bacteria isolated from natural environments or culture conditions, bacterial synergy is often unexplored or under-reported. In this study, we isolated bacterial strains from the local eutrophic river canal and evaluated their suitability for precipitating calcium carbonate polymorphs. We identified two distinct bacterial isolates with superior urea degradation ability (conductivity method) using partial 16 S rRNA gene sequencing. Molecular identification revealed that they belong to the *Comamonas* and *Bacillus* genera. Urea degradation analysis was performed under diverse pH (6,7 and 8) and temperature (15 °C,20 °C,25 °C and 30 °C) ranges, indicating that their ideal pH is 7 and temperature is 30 °C since 95% of the urea was degraded within 96 h. In addition, we investigated these strains individually and in combination, assessing their microbially induced carbonate precipitation (MICP) in silicate fine sand under low (14 ± 0.6 °C) and ideal temperature 30 °C conditions, aiming to optimize bio-mediated soil enhancement. Results indicated that 30 °C was the ideal temperature, and combining bacteria resulted in significant (*p* ≤ 0.001) superior carbonate precipitation (14–16%) and permeability (> 10^− 6^ m/s) in comparison to the average range of individual strains. These findings provide valuable insights into the potential of combining ureolytic bacteria for future MICP research on field applications including soil erosion mitigation, soil stabilization, ground improvement, and heavy metal remediation.

## Introduction

Microbially Induced Calcium Carbonate Precipitation (MICP) is an environmentally friendly process that utilizes microorganisms to instigate the formation of calcium carbonate (CaCO_3_) polymorphic minerals from soluble calcium ions and bicarbonate ions, often in conjunction with urea or nitrogen sources (Lv et al. 2023). This process has garnered increasing interest from researchers in recent years due to its many potential uses. MICP offers environmental advantages over cement by utilizing microbial processes to precipitate calcium carbonate, reducing energy-intensive production and lowering carbon emissions. Unlike cement, MICP operates at ambient conditions, emitting fewer greenhouse gases and promoting the use of naturally occurring materials and microbial catalysts, allowing the reduction in the carbon footprint of construction (An et al. 2024). With applications ranging from soil stabilization to groundwater remediation and via biocementation the repair of construction materials, MICP has emerged as a promising environmentally friendly technology for addressing engineering challenges (Liu et al. 2020; Rajasekar et al. 2021b; Bu et al. 2022).

Central to the MICP phenomenon is the pivotal role of microorganisms, notably bacteria and fungi. These microorganisms serve as catalysts since their metabolic capabilities bring about essential chemical transformations that facilitate the precipitation of calcium carbonate minerals (Seifan and Berenjian 2019; Zhou et al. 2023). Microorganisms effectively augment the local concentration of calcium and carbonate ions by releasing organic acids, urease, or ammonium during their metabolic activities, creating an environment conducive to calcium carbonate nucleation and subsequent crystal growth.

To carry out MICP, urease is the most prominent enzyme. Urease catalyzes the hydrolysis of urea into ammonia (NH_3_) and carbon dioxide (CO_2_). Subsequently, ammonia interacts with calcium ions (Ca^2+^) in the surrounding environment, leading to the formation of calcium carbonate (CaCO_3_) (Krajewska 2018). Remarkably, certain ureolytic bacteria, such as *Sporosarcina pasteurii*, have demonstrated the capability to significantly enhance MICP efficiency by ensuring a continuous supply of urease (Omoregie et al., 2017, Chen et al. 2022; Lapierre et al., 2020). Several other bacterial species and strains have also been reported in recent years for their ability to precipitate calcium carbonate polymorphs through urease activity (Oualha et al. 2020; Liu et al. 2019; Peng et al. 2016). These bacterial species sustain precipitation, laying the foundation for developing stable and compacted cementitious matrices. Recent MICP advancements include novel bacterial strains, adaptable to diverse environments, and treatments on varied soil types, including calcareous, laterite, and clay soils, demonstrating the versatility of this technique and its relevance in addressing modern challenges (Chen et al. 2023; Ghorbanzadeh et al. 2020; Li et al. 2023b).

Traditionally, researchers have concentrated their efforts on individual bacterial strains, such as *Sporosarcina pasteurii* or native bacterial strains, as the focal point of MICP studies. However, recent investigations have delved into a more collaborative approach: bacterial consortia (Sharma et al., 2022). This innovative approach focuses on the synergy among different bacterial strains within a consortium. The idea is to utilize potential urease-positive strains, each with unique levels of urease activity, to cooperate within a consortium. Certain strains may exhibit high urease activity yet fall short in calcium carbonate precipitation proficiency, while others display lower urease activity but excel in precipitate formation. By collaborating, these bacterial strains achieve more efficient conversion of urea into ammonia, producing superior amounts of calcium carbonate precipitates (Zhu and Dittrich 2016; Wang et al. 2024b; He et al. 2023) and allowing precipitation to occur effectively across various environmental conditions. This cooperative action transforms into accelerated urea hydrolysis and ammonia production, promoting the formation of calcium carbonate crystals within the soil matrix.

The success of MICP treatments hinges significantly on manipulating and maintaining pH levels, especially in demanding environments such as sand (Almajed et al. 2021; Mujah et al. 2017; Oualha et al. 2020; Mahawish et al. 2018). pH control ensures the preservation of conditions that favor efficient MICP. Temperature plays a pivotal role in dictating MICP’s effectiveness. Temperature can influence the metabolic activity of ureolytic bacteria, subsequently impacting the kinetics of urea hydrolysis and calcium carbonate precipitation (Helmi et al. 2016; Sun et al. 2019). Cooperative interactions within a bacterial consortium become particularly consequential here. Different bacterial strains may exhibit varying temperature tolerances and growth rates. Specific strains thrive at elevated temperatures, while others exhibit heightened activity in lower-temperature environments. While promising, MICP encounters challenges in pH and temperature control, scalability, and cost-effectiveness. Maintaining optimal pH conditions is crucial for calcium carbonate precipitation, while temperature variations affect microbial activity. Scalability issues arise due to cost concerns and the challenges of large-scale implementation. Recognizing these obstacles is essential for understanding MICP’s practical implications and underscores ongoing research’s need to address these limitations effectively.

MICP’s potential applications are broad, from enhancing the load-bearing capacity of foundations in construction to mitigating soil erosion and stabilizing slopes in geotechnical projects. This microbial geochemical process offers a sustainable alternative to conventional cement-based solutions, reducing carbon emissions and environmental impact (Achal and Kawasaki 2016; Namdar-Khojasteh et al. 2022; Wilkinson and Rajasekar 2023). The biocementation process can also be manipulated to meet specific project requirements by adjusting microbial strains to environmental conditions. In slope stabilization, MICP-treated soils exhibit increased shear strength and reduced erosion susceptibility, mitigating the risk of landslides and slope failures (Wang et al. 2024b; Hang et al. 2023). Furthermore, MICP enhances the load-bearing capacity of foundations by reinforcing soil matrices and reducing settlement, thereby improving the stability and longevity of structures (Sun et al. 2024). Additionally, MICP contributes to soil erosion control by cementing soil particles and enhancing cohesion, preventing erosion and sediment runoff (Wang et al. 2024b; Gitanjali et al. 2024). These practical applications underscore the tangible benefits of MICP in real-world geotechnical engineering projects. Hence, there is a potential for the diverse strains to collaborate within a consortium; they create a dynamic and adaptable force capable of operating efficiently across a broad temperature range. This adaptability represents a considerable advantage in geotechnical applications, where temperature variations can be substantial. Despite significant advancements, gaps persist in our understanding of MICP mechanisms and their application across diverse environmental conditions. Further research is needed to elucidate the complex interactions between microbial communities and environmental factors, optimizing MICP efficiency and reliability. Moreover, exploring the scalability and cost-effectiveness of MICP treatments on a larger scale is essential for widespread adoption in engineering practice (Zhang et al. 2023). Addressing these knowledge gaps will facilitate the development of more robust and versatile MICP techniques, driving innovation and expanding the application domain of this sustainable technology.

Cooperative bacterial interactions within MICP represent a promising frontier in this innovative geotechnical technique. A comprehensive understanding of these interactions holds the key to unlocking the full potential of MICP in enhancing soil and sand properties and stabilization. By investigating the synergistic effects of bacterial consortia in MICP across different temperatures and cementation conditions, this study highlights microbial interactions and their practical implications for geotechnical applications. Specifically, elucidating the cooperative mechanisms underlying MICP and evaluating the feasibility of these mechanisms for sustainable soil stabilization and construction practices. Through studying pH and temperature variables, this research endeavors to provide valuable insights into optimizing MICP techniques and advancing the field of microbial geotechnics.

## Materials and methods

### Bacterial isolation and urea determination

The following protocol by Rajasekar et al. (2018) was used to isolate 15 bacteria from a local eutrophic canal using Nutrient agar (10 g/L peptone, 3 g/L beef extract, 5 g/L NaCl, and 15 g/L agar) (hopebio™, China) For this study, bacterial isolates were obtained using the following procedure. Water samples with serial dilutions were spread onto a Petri plate nutrient agar and incubated at 30 °C for 24 h until visible colonies were obtained. The bacterial isolates were purified by repeated streaking and then transferred into nutrient broth (hopebio™, China). The eutrophic canal is surrounded by vegetation at the university campus (32°12′18.9″N 118°43′23.4″ E). The physicochemical parameters of the water sample are as follows: pH was 7.8, the temperature was 23.4 °C, Total Nitrogen was 3.6 mg/L, and Total phosphorus was 1.1 mg/L. The strains were tested for the urease enzyme using the Christensen urea-Agar method (Leeprasert et al. 2022). The initial urease activity of the urease-positive strains was determined using a conductivity method (Harkes et al. 2010; Konstantinou et al. 2021). This method is beneficial when calcium ions are absent in the substrate medium. The methodology for assessing urease activity involved introducing 9 mL of a urea solution (1.11 M) to 1 mL of a bacterial suspension at an OD_600_ of 1 at 25 °C. Subsequently, an electrical conductivity meter was utilized to monitor alterations over a specified time interval, and the quantity of hydrolyzed urea (Eq. 1) was measured using the average over 5 min.

1 $$Urea \,Hydrolysed \,\left(mM\right) = Conductivity \,\left(mS\right) * 11.11$$

Two high-performing bacterial strains were chosen from the conductivity method for the sand cementation experiment.

### Urea degradation experiment

The urea degradation of the urease-positive strains was determined by a solution containing 20 g/L Urea (SCP™, China) and nutrient broth (10 g/L peptone, 3 g/L beef extract, 5 g/L NaCl) (hopebio™, China). The nutrient broth was prepared separately under sterile conditions and autoclaved at 121 °C for 15 min. After the nutrient broth cooled down, Urea was added using filter sterilization to maintain high sterile conditions and avoid cross-contamination. The conical flasks containing 20 g/L urea and nutrient broth were placed in an environmental shaker incubator that maintained 150 rpm for 96 h of the experiment. The experimental setup is shown in Table 1. The initial pH of each experiment was obtained by adding either HCl or NaOH. The urea degradation test was performed following the protocol established and applied by (Zhang et al. 2022). The urea concentration was determined by p-dimethyl amino benzaldehyde (PDAB) colorimetry (Knorst et al. 1997). A 1 mL PDAB reagent was added to 10 mL of diluted sample, and the absorbance was recorded. A control sample containing no bacteria was included in the experiment. The absorbance was measured at 420 nm using a 754 UV-Vis spectrophotometer (JINGHUA Instruments Co., Ltd, Shanghai, China). All experiments were conducted in triplicate.

**Table 1:** Experimental setup showing the chemical and environmental conditions for the urea degradation experiment

Sample	Starting pH	Total volume	Temperature (°C)
Control^*^	6	100 mL	15
B2^*^	6	100 mL	20
B11^*^	6	100 mL	25
B2&B11^*^	6	100 mL	30
Control^*^	7	100 mL	15
B2^*^	7	100 mL	20
B11^*^	7	100 mL	25
B2&B11^*^	7	100 mL	30
Control^*^	8	100 mL	15
B2^*^	8	100 mL	20
B11^*^	8	100 mL	25
B2&B11^*^	8	100 mL	30

^*^= conducted in triplicates. B2 = *Bacillus Subtilis* HMZC1; B11 = *Comamonas fluminis* HMZC

### 16s rRNA sequencing

The genetic identification of the chosen microorganisms involved comparing their 16 S rRNA gene sequences with sequencing performed by Nanjing Springen Biotechnology Co., Ltd. To amplify the 16 S rRNA gene of these strains, we employed PCR with the universal primers 27 F (5’-AGAGTTTGATCCTGGCTCAG-3’) and 1492R (5’-CTACGGCTACCTTGTTACGA-3’) targeting both ends of the gene (Frank et al. 2008). The PCR reaction comprised one µL of DNA, 2 × 25 µL PCR mix buffer from Springen (China), one µL of each primer at a concentration of 10 µL, and 22 µL of double-distilled water, totaling 50 µL. The PCR program commenced with a 5-minute pre-denaturation step at 95 °C, followed by 35 cycles: 95 °C for 30 s, 55 °C for 30 s for pre-annealing, 72 °C for 1 min and 30 s for annealing, and a final extension at 72 °C for 7 min. Subsequently, the PCR products underwent recovery using the Springen Agarose Gel Magnetic Bead Method DNA Recovery Kit (MD003-100). Using 100 nanograms of purified DNA template, Sanger sequencing was employed on the ABI 3730-XL Genetic Analyzer (Thermo Fisher Scientific in Waltham, MA, USA). The nucleotide sequences were edited with DNAstar Lasergene software (version 7.1). We aligned the sequencing data with relevant 16 S rRNA gene sequences in the National Center for Biotechnology Information (NCBI) GenBank database to determine the microbial identity through a BLAST search. The bacterial strains chosen in this study were B2 and B11, and their accession numbers are OQ826692 (similarity index 86.48% and 1229 base pairs) and OQ826707 (similarity index 98.39% and 1198 base pairs). B2 = *Bacillus Subtilis* HMZC1; B11 = *Comamonas fluminis* HMZC.

### Sand cementation experiment

We assessed precipitated calcium carbonate directly within a 50 mL transparent polypropylene (PP) centrifuge tube. To ensure the integrity of our experimental setup and mitigate any potential contamination, these tubes were carefully placed inside 250 mL beakers. Non-compacted siliceous sand (50 g) (Table 2) was carefully introduced into these tubes, equipped with a 20 mm drainage aperture at their base to facilitate fluid flow/drainage. To prevent the inadvertent spillage of sand and liquids, a layer of transparent tape was carefully applied at the base of each tube. A filter paper was placed at the bottom of the tube to ensure no sand leaked during cementation solution replacement. 40 ml of cementation solution was added slowly using a sterile syringe at the top of the tube.

**Table 2:** The properties of the sand used in this study

Properties	Values
Specific gravity	2.65 g/cm^3^
Coefficient of uniformity	1.9
Composition	SiO_2_
Shape	Round or Angular
Permeability	3.2 × 10^−3^ m/s
e_max_	0.425 mm
e_min_	0.22 mm
Coefficient of curvature	1.14

Before commencing the experiments, all tubes and bottles were thoroughly washed with a 70% ethanol solution to maintain an uncontaminated experimental environment. The cementation solution was recirculated every 12 h. Calcium chloride and urea were prepared in separate bottles using double distilled water. A filter sterilization technique was implemented to combine calcium chloride and urea solution before adding the nutrient broth, which contains the bacteria. The pH was adjusted to 7.0 using 1 M NaOH (if it is acidic) or 1 M HCl (if it is alkaline). The cementation solution was replaced every 48 h; the contents of the cementation solution are shown in Table 3. The sand tubes received three cycles of treatment. A control sample containing no bacteria was included in the experimental study. All experiments were conducted in triplicate.

**Table 3:** The cementation experiment protocol

Bacteria	Initial pH	Temperature	Calcium chloride	Urea	Nutrient substrate
B2	7.0	14 ± 0.6 °C	0.5 M	0.5 M	Nutrient broth + Bacteria (OD = 1.0)
B11	7.0	14 ± 0.6 °C	0.5 M	0.5 M	Nutrient broth + Bacteria (OD = 1.0)
B2&B11	7.0	14 ± 0.6 °C	0.5 M	0.5 M	Nutrient broth + Bacteria (OD = 1.0)
Control	7.0	14 ± 0.6 °C	0.5 M	0.5 M	Nutrient broth + Bacteria (OD = 1.0)
B2	7.0	30 °C	0.5 M	0.5 M	Nutrient broth + Bacteria (OD = 1.0)
B11	7.0	30 °C	0.5 M	0.5 M	Nutrient broth + Bacteria (OD = 1.0)
B2&B11	7.0	30 °C	0.5 M	0.5 M	Nutrient broth + Bacteria (OD = 1.0)
Control	7.0	30 °C	0.5 M	0.5 M	Nutrient broth + Bacteria (OD = 1.0)

B2 = Bacillus Subtilis HMZC1; B11 = Comamonas fluminis HMZC

### Permeability

Permeability assessments were conducted through a falling head procedure, as described in (BSI 2019). The coefficient of permeability (K) was calculated using the equation:

$$K=2.303\times \left[\frac{aL}{A({t}_{f}-{t}_{i})}\right]\times {log}_{10}\frac{{h}_{1}}{{h}_{2}}$$

Where a is the area of the inlet, L is the distance between the two measuring points. A is the sample area, (t_f_-t_i_) is the increment of time between two readings, h_1_ is the head of water above outlet elevation at time ti, and h_2_ is the head above outlet elevation at time t_f_. (Rajasekar et al. 2021a). This procedure involved measuring the water flow rate between two designated marks in the column above the sand layer. After completing the sand cementation experiment, all sand tubes underwent thorough flushing with deionized (DI) water to eliminate any residual chemicals present within the coarse sand’s pore throats. Subsequently, the sand specimens were saturated with water before commencing the permeability test. This saturation process involved percolating water through the coarse sand to expel any remaining air within the pore matrix.

### Scanning electron microscopy

A thorough procedure was followed for the sample preparation intended for SEM analysis, which was performed using the thermo-field emission FEI Quanta 400FEG microscope (Thermo Fisher Scientific, Waltham, MA, USA) at magnifications ranging from 200× to 50,000×. The SEM imaging was conducted at an accelerating voltage of 20 kV. Working distance (WD) ranged from 12.9 to 13.9 mm. The acceleration voltage and WD are shown in the corresponding SEM images at the bottom left corner. Each sand tube experiment sample underwent a preliminary step to eliminate any potential moisture content. Specifically, these sand samples were carefully placed inside an oven and subjected to a temperature of 48℃ over three days. This deliberate drying ensured that the samples were completely moisture-free, facilitating accurate analysis. Subsequently, various samples were affixed onto the electron microscope stubs using conductive adhesive. Before SEM analysis, a gold layer was sputtered onto the sample’s surface. The area of interest was based on the visibility and clarity of the biocementation and mineral morphology that could be used to explain the MICP process.

### X-ray diffraction spectroscopy

For X-ray diffraction (XRD) (SmartLab (9) diffractometer (Rigaku, Japan) with an ultra-high speed detector (40 kV and 150 mA) scanning from 3° to 90° at a step rate of 8°/min, each sand sample taken from the sand tube experiment had to be ground entirely and placed in the oven for three days at 48℃ to remove all the possible moisture content. After drying, the sand was placed in a sterilized pestle and mortar and ground to a powder for XRD analysis. We employed the Match3! Software to identify and characterize the calcium carbonate precipitates. Uploading the XRD file onto the software helps detect relevant calcium carbonate polymorphs by comparing the peaks against the International Centre for Diffraction Data and Crystallography open database.

### Carbonate analysis

The carbonate content was determined by following the protocol discussed by Rajasekar et al. (2021a). The procedure is as follows: weigh the soil sample of 10 g (± 0.001 g) into a 250 mL Erlenmeyer flask: use a volumetric pipette; add 20 mL of standardized 1 N HCl to the flask; cover the Erlenmeyer flask with a watch glass and boil the soil-acid mixture for 5 min; and add 50–100 mL deionized water using a graduated cylinder. After it has cooled down, add 2 or 3 drops of phenolphthalein indicator. Titrate the solution with 1 N NaOH solution while swirling the flask and finally take the reading when a faint pink color develops.

$${\text{C}\text{a}\text{C}\text{O}}_{3}\text{e}\text{q}\text{u}\text{i}\text{v}.,\%=\left(\frac{{\text{V}}_{HCl}{\text{N}}_{HCl}-{\text{V}}_{NaOH}{\text{N}}_{NaOH}}{\text{g}\text{r}\text{a}\text{m}\text{s} \,\text{o}\text{f} \,\text{s}\text{o}\text{i}\text{l}}\right)\times 0.05\times 100$$

Where V_HCl_ N_HCl_ and V_NaOH_ N_NaOH_ are the volume and normality of HCl and NaOH, respectively. We analyzed the top and bottom half of the sand column to identify calcium carbonate cementation and its variation between bacteria.

### Statistical analysis

The data are presented as means along with their corresponding standard deviations. Statistical analyses, including analysis of variance (ANOVA) and other calculations, were conducted using GraphPad Prism 10 software, with a confidence interval of 95%. Error bars in the figures, representing the variability around the mean, were generated using Microsoft Excel 2019.

## Results and discussion

### Urea degradation

**Fig. 1:**
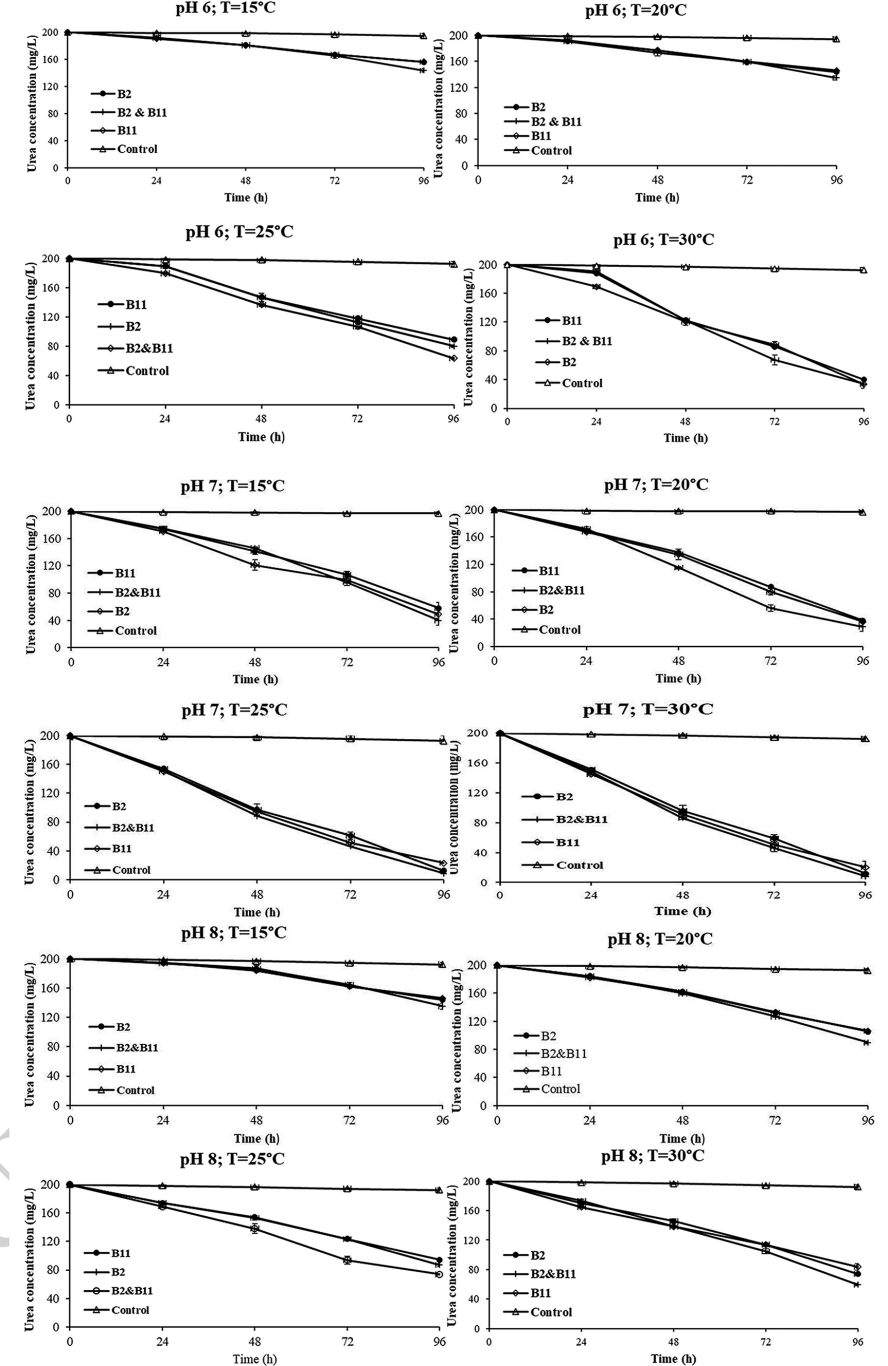
Urea degradation experiment by control, B2, B11 and B2&B11. B2 = *Bacillus Subtilis* HMZC1; B11 = *Comamonas fluminis* HMZC

The success and efficiency of MICP are highly dependent on several key factors, including pH, temperature, and the unique biochemical characteristics of different bacterial strains. Two of the 15 bacterial strains were chosen for their superior urease activity. In order to determine their ability to degrade urea to precipitate carbonate, a series of experiments at different pH and temperature were conducted to optimize for higher calcium carbonate precipitations. The results from Fig. 1 show that at pH 7 and 30 °C, the bacteria performed significantly better compared to other pH and temperature ranges. Other studies have also identified these as optimum urease activity for superior carbonate precipitation (Zhou et al. 2023; Sun et al. 2021; Bai et al. 2021; Naveed et al. 2020). Roughly 40–60% of the urea was degraded at 15 and 20 °C at pH 6 and 8 compared to pH 7; this indicates that these bacterial strains prefer neutral pH and higher temperatures. Previous studies also found that native ureolytic bacterial strains perform better at a pH of 6.5–7.5 (Seifan and Berenjian 2019; Cheng et al. 2014). *Sporosarcina pasteurii* and *Bacillus megaterium* are outliers concerning pH, as they exhibit superior urease activity at pH 8 to 9 (Chen et al. 2022; Sun et al. 2019; Dong et al. 2023).

In this study, the temperature significantly affected urea degradation compared to pH. Urea degradation at 15 and 20 °C were significantly lower when compared to 25 and 30 °C, irrespective of the starting pH. This indicates that even if the pH is slightly acidic or alkaline, the bacteria can degrade urea through urease activity if the temperature is within the correct range (Helmi et al. 2016). Previous research on urease activity has confirmed that higher temperatures generally accelerated urea hydrolysis and ammonia production due to the temperature-dependent kinetics of enzymatic reactions (Krajewska 2018; Zhao et al. 2014; Konstantinou et al. 2021). The results from those studies have found that extreme or non-optimal temperatures can denature urease enzymes, rendering them inactive. Previous studies have reported a significant reduction in urease activity when temperatures go higher than 30 °C and lower than 20 °C (Anbu et al. 2016; Rajasekar et al. 2021b), thus supporting our data that 25 °C and 30 °C are the ideal temperatures for most ureolytic bacteria. Therefore, precise temperature control is imperative to maintain optimal urease activity in the MICP processes. This observation highlights the significance of considering the temperature requirements of selected bacterial strains when implementing MICP for practical applications. B2 performed better than B11 in degrading urea under diverse environmental conditions, suggesting that B2 is more resistant and adaptable to changing environmental conditions. The bacteria B2 belongs to the *Bacillus* genera, which has been studied extensively for MICP, and its versatility is well documented (Namdar-Khojasteh et al. 2022; Oualha et al. 2020; Sun et al. 2019; Raut et al. 2014). Our study found that *Comomonas* genera (bacteria B11) also possesses the urease enzyme and its ability to precipitate carbonates. *Comomonas* genera are well known for their ability to sequester CO_2_ to precipitate calcite (Okyay et al. 2016).

Interestingly, we found that when B2 and B11 bacteria were combined, they had superior (10–22%) urea degrading ability compared to B2 alone. The superior urea-degrading ability in bacterial consortia compared to individual strains (B2 and B11) highlights the potential for microbial synergy to enhance MICP processes. This phenomenon suggests that the combined metabolic activities of different bacterial species may lead to more efficient urea hydrolysis and carbonate precipitation, thereby amplifying the effectiveness of biocementation strategies. This result indicates that bacterial synergy could potentially lead to the application of MICP in soil by biostimulation rather than bioaugmentation (Mahanty et al. 2013). The mechanisms underlying bacterial synergy in MICP processes are multifaceted and may involve various factors such as metabolic cooperation, niche complementarity, and nutrient sharing. Different bacterial species can complement each other’s metabolic pathways in a microbial consortium, enhancing overall metabolic efficiency (Zhou et al. 2023). In a synergetic relationship, one species may be superior in urea hydrolysis; another may be more efficient in mineral nucleation, resulting in superior and efficient MICP performance. Previous studies on synergetic relationships usually focused on ureolytic bacteria with biochar (Xu et al. 2023), fiber (Choi et al. 2019), zeolite (Jafarnia et al. 2020), or rubber (Feng et al. 2022). Since most of the applications for MICP are in soil stabilization, crack healing, and preventing erosion, it would be highly encouraging if the bacterial synergistic relationships are performed in the presence of a carrier material (fiber, zeolite, or rubber) under challenging environmental conditions to provide long term sustainable solutions (Namdar-Khojasteh et al. 2022; Leeprasert et al. 2022; Pungrasmi et al. 2019).

### Sand cementation experiments

**Fig. 2:**
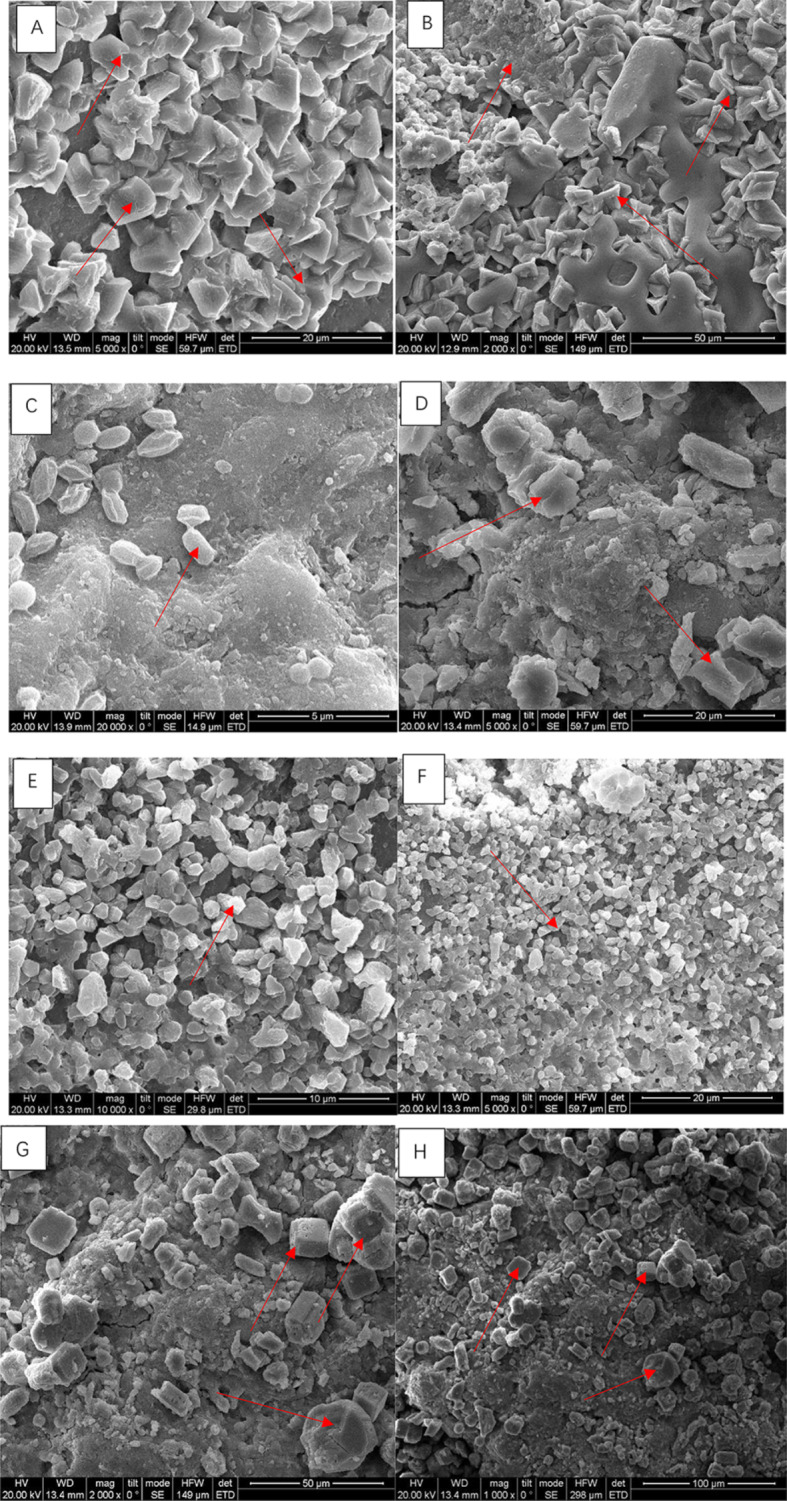
Scanning electron microscope images of CaCO_3_ on the surface of the sand particles. A = B2-R; B = B11-R, C, D = B2 & B11- R; E = B2-30 °C, F = B11-30 °C, G,H = B2 & B11- 30 °C. B2 = *Bacillus Subtilis* HMZC1; B11 = *Comamonas fluminis* HMZC

**Fig. 3:**
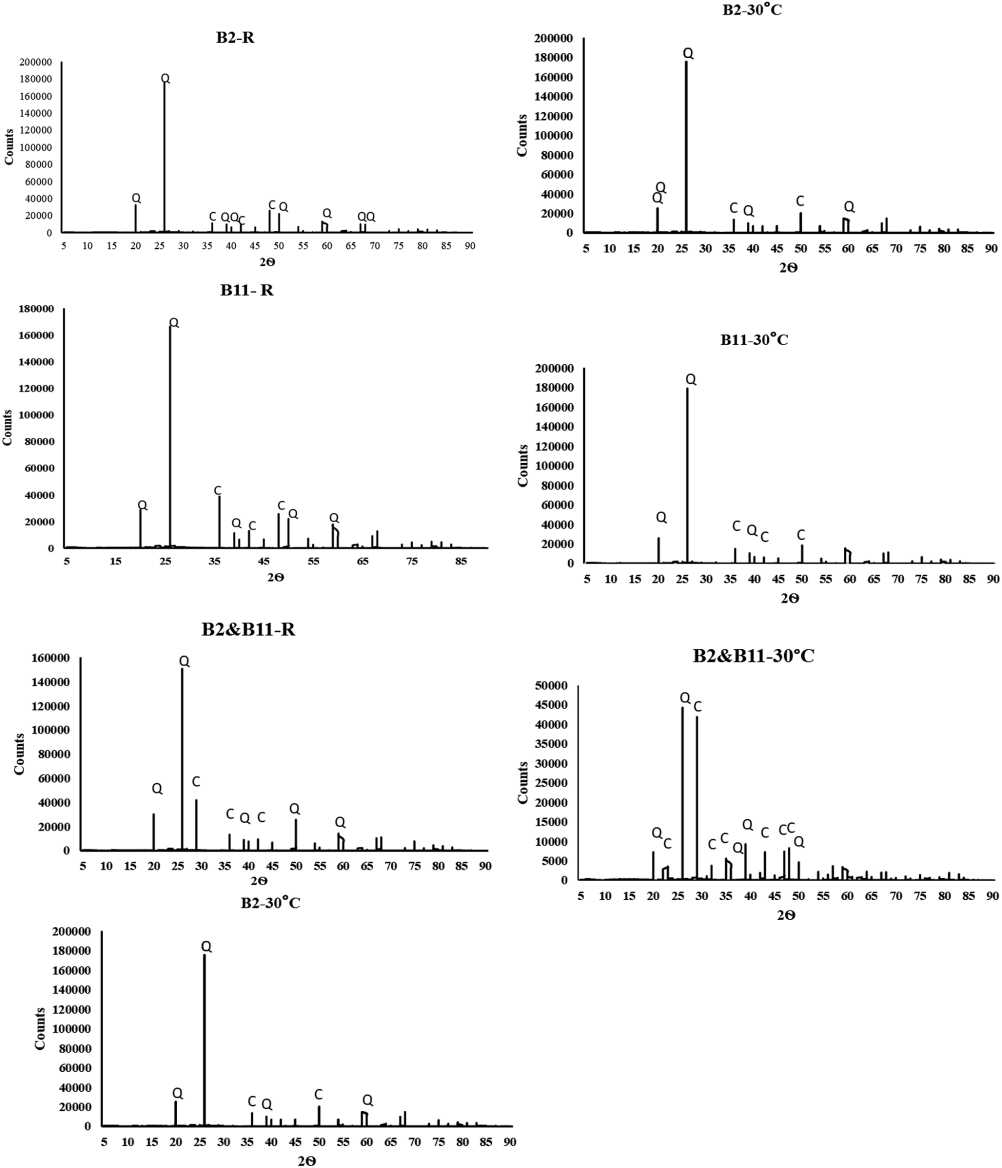
X-ray diffraction graphs that show calcite precipitation by B2-R, B11-R, B2 & B11-R and B2-30 °C, B11-30 °C, B2 & B11-30 °C, respectively. B2 = *Bacillus Subtilis* HMZC1; B11 = *Comamonas fluminis* HMZC

The bacterial strains were applied to sand samples using a bioaugmentation approach, where bacterial suspensions were evenly distributed over the sand surface. The sand samples were then incubated under controlled conditions to allow bacterial colonization and urease activity, facilitating calcium carbonate precipitation. Calcite precipitation was assessed using X-ray diffraction (XRD) analysis to identify mineral phases and scanning electron microscopy (SEM) to visualize crystal morphology and distribution. Multiple studies have used these methods to provide insights into the effectiveness of bacterial strains in promoting sand cementation and calcium carbonate precipitation (Spencer et al. 2023; Zeitouny et al. 2023). The ability of the bacterial strains to perform in sand was assessed at two different temperatures, 14 ± 0.6 °C and 30 °C. Both temperature and bacterial combinations affect the shape of calcite crystals (Fig. 2A–H). Previous studies have identified that bacteria influence and dictate the crystals’ shape (Li et al. 2018; Rui and Qian 2022). Crystal shapes such as rhombohedral, spherical, and cylindrical play a crucial role in understanding the formation and stability of calcite crystals during sand cementation experiments. Rhombohedral crystals have a characteristic diamond-like shape with six faces, while spherical crystals are round and lack distinct edges or corners. Cylindrical crystals are elongated with parallel sides and rounded ends. Several previous studies have observed these shapes in calcite crystals during MICP processes (Wang et al. 2024a; Li et al. 2023b; Zhao et al. 2024). We found that a combination of B2 & B11 at 14 ± 0.6 °C produced cylindrical crystals (Fig. 3C & D), while at 30 °C, they precipitated rhombohedral structures. Our study identifies temperature as an influencer on the shape of the formed crystal (Fig. 3A and B vs. E and F); temperature may help to stabilize the crystals. The crystal morphology for B2-R and B11-R are quite similar; their shape is neither rhombohedral nor spherical or cylindrical. However, the crystal shapes for B2-30 °C and B11-30 °C were cylindrical and spherical (Wang et al. 2024a). The combination of B2&B11 also yielded differences in crystal shape (Fig. 3C and D vs. G and H). The morphology of B2& B11-R are irregular and angular, but B2 & B11-30 °C were observed to be rhombohedral and more rounded. Commonly, clusters of calcite crystals are observable, adhering to the surface of sand particles. This phenomenon fosters a cohesive connection between the calcite crystals and the individual grains of sand. Previous studies have identified that spherical and rhombohedral are often the most stable forms of calcite (Rahman et al. 2020; Kim et al. 2018; Ye et al. 2023). Furthermore, previous SEM observations have revealed a close agglomeration of rhombohedral and blocky calcite structures when subjected to the influence of a calcium chloride/urea cementation solution. (Naveed et al. 2020; Sun et al. 2019; Erdmann and Strieth 2023). Thus, temperature also plays a role in determining the shape of the crystals and their application in soils (Bu et al. 2022; Peng and Liu 2019).

We observed calcite precipitation by all bacteria at 14 ± 0.6 °C and 30 °C (Fig. 2). The analysis revealed that quartz (SiO_2_) constituted the primary component of the original sand, with a prevailing crystal structure. After quartz, we found calcite dominant in the cementation sand for B2, B11, and “B2&B11”. We observed that “B2&B11” at 30 °C displayed a peak pattern at 29.3°, which was not observed for any other samples; this emphasizes the synergetic calcite precipitation and potentially a structural prominence within the sand. Previous studies also observed calcite in their sand experiments (Lv et al. 2023; Sharma et al. 2021; Zhao et al. 2024; Ma et al. 2022).

Cementation of sand particles is pivotal for stabilizing soil, mitigating erosion, sealing cracks, and ground improvement. This process involves the precipitation of calcium carbonate on the surfaces of sand particles, which plays a significant role in forming cement connections between sand particles. Assessing calcium carbonate precipitation and measuring permeability serve as indicators for gauging the effectiveness of sand particle cementation. Permeability refers to the ability of a material, such as sand, to allow fluids to pass through it (BSI 2019). In the context of sand particle cementation, permeability is a critical parameter for assessing the effectiveness of cemented sand structures. The permeability results provide valuable insights into the effectiveness of MICP processes for soil stabilization and erosion control. Lower permeability values indicate tighter packing of sand particles and reduced pore spaces, resulting in improved soil stability and erosion resistance. Changes in permeability affect the overall performance of cemented sand structures, with lower permeability corresponding to greater soil stability and durability. Understanding the implications of permeability results is essential for optimizing MICP techniques and designing effective soil stabilization strategies (Chen et al. 2023; Pacheco et al. 2022).

**Fig. 4:**
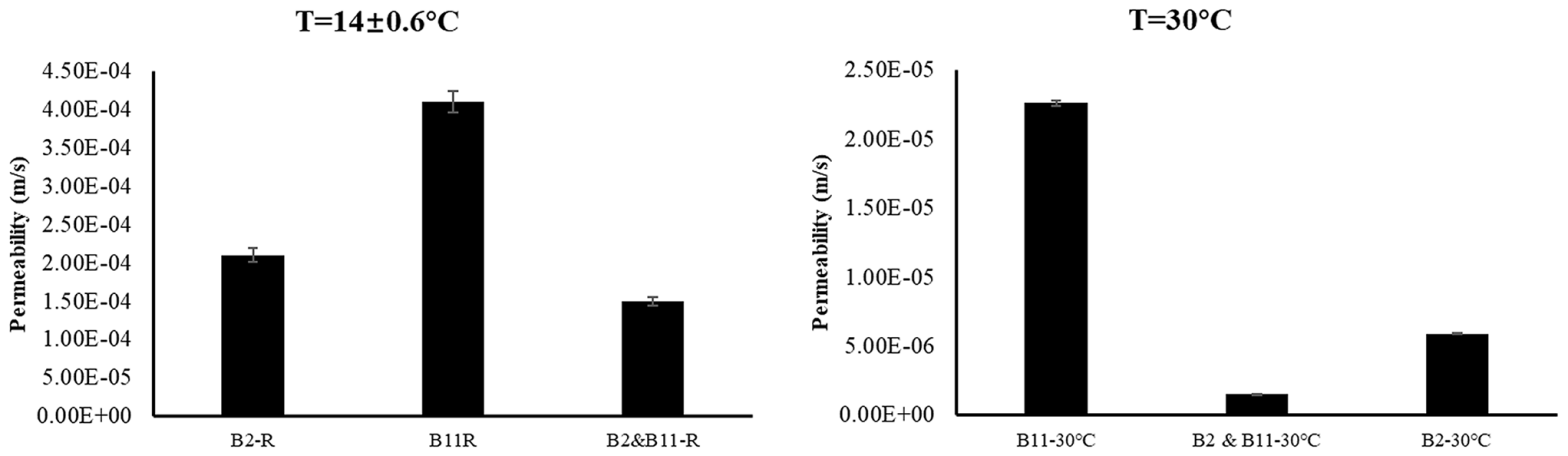
Permeability values of B2, B11, B2 & B11 at different temperatures. B2 = *Bacillus Subtilis* HMZC1; B11 = *Comamonas fluminis* HMZC

This study showed a notable difference in permeability between the two bacterial strains, B2 and B11, under varying temperature conditions. Specifically, at 30 °C, both strains demonstrated superior performance compared to the conditions at 14 ± 0.6 °C (Fig. 4), and they were statistically significant (Table 4). This observation aligns with previous studies, and our study highlights the enhanced urease activity associated with higher temperatures in sand cementation research (Cheng et al. 2015; He et al. 2020; Murugan et al. 2021; Chen et al. 2023). The significant outcome of our study was the achievement of a permeability rate in the range of 10^− 6^ m/s with strain B2 at 30 °C, as depicted in Fig. 4. This result underscores the efficacy of cementation between sand particles. Notably, similar findings have been reported in previous studies where individual bacteria were employed to improve permeability in the sand (Liu et al. 2020; Namdar-Khojasteh et al. 2022; Spencer et al. 2023; Ugur et al. 2024). The B2 & B11-30 °C combination yielded the best permeability result with 1.54 × 10^− 6^ m/s compared to all the other bacterial samples. The relationship between permeability, urease activity, and calcium carbonate content is well-established (Sharma et al. 2021; Seifan and Berenjian 2019; Gebru et al. 2021). Urease activity catalyzes urea hydrolysis, yielding carbonate ions crucial for calcium carbonate precipitation. As calcium carbonate forms, pore spaces in the soil matrix diminish, reducing permeability. Higher urease activity correlates with increased carbonate ion availability and subsequent calcium carbonate content, enhancing soil cementation and decreased permeability. This relationship underscores the pivotal role of urease activity in biocementation processes, where the interplay between urea hydrolysis, calcium carbonate precipitation, and permeability reduction influences soil stabilization and erosion control. Moreover, the variance in size and morphology between bacterial strains B2 and B11 may also contribute to their superior cementation performance. It is conceivable that the combined consortia, comprising bacterial cells of diverse shapes and sizes, can effectively penetrate and colonize a broader range of pore spaces within the sand matrix. This extensive colonization enables thorough coverage of sand particles and facilitates more extensive calcium carbonate precipitation, leading to improved cementation and permeability reduction (Almajed et al. 2021; Tang et al. 2020). One potential mechanism underlying synergistic effects is metabolic cooperation among bacterial species. Different bacterial strains may possess complementary metabolic pathways, utilizing available resources and substrates. For example, one bacterial species may excel in urea hydrolysis, releasing carbonate ions crucial for calcium carbonate precipitation, while another species may specialize in mineral nucleation or crystal growth (Tiwari et al. 2021; Wang et al. 2022). These bacterial species can synergistically enhance calcium carbonate precipitation and sand cementation efficiency.

**Table 4:** Permeability between treatment groups. Groups with the same letter are not significantly different

Sample type	Mean ± SD
B2-R	2.15E-04 ± 8.99E-06b
B11R	4.24E-04 ± 1.43E-05a
B2&B11-R	1.54E-04 ± 5.72E-06c
B11-30℃	2.25E-05 ± 2.16E-07d
B2 & B11-30℃	1.53E-06 ± 1.63E-08d
B2-30℃	5.88E-06 ± 2.45E-08d

SD = Standard Deviation

**Fig. 5:**
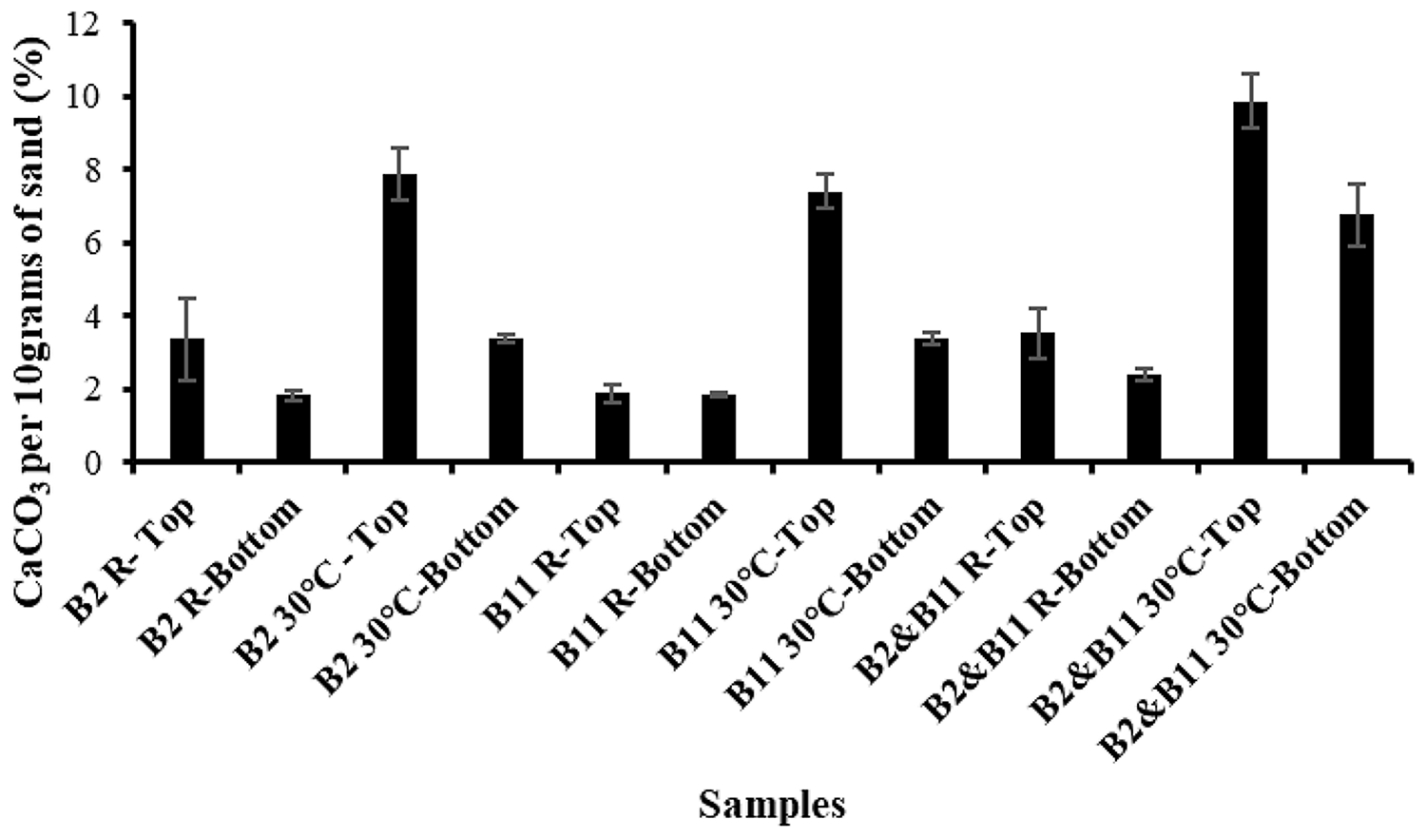
Calcium carbonate precipitation (%) values of B2, B11, B2 & B11 at *R* = 14 ± 0.6 °C and 30 °C. B2 = *Bacillus Subtilis* HMZC1; B11 = *Comamonas fluminis* HMZC

Our findings consistently indicate that B2 and the combination of B2 and B11 at 30 °C outperform other calcium carbonate content conditions, as demonstrated in Fig. 5. We found that bacteria at 30 °C had statistically significant precipitation compared to their counterparts, indicating that temperature plays a significant role in calcium carbonate precipitation (Table 5). Previous studies have also found temperatures between 25 and 35 °C ideal for calcium carbonate precipitation due to superior bacterial growth and metabolic activity (Rui and Qian 2022; Rollakanti and Srinivasu 2022; Erdmann and Strieth 2023). The observed trend of enhanced cementation in the top half of the sand column aligns with previous research findings, suggesting a common phenomenon attributed to bacterial growth dynamics and oxygen availability within the column (He et al. 2020; Chu et al. 2012; Fronczyk et al. 2023). We believe this could be due to the bacteria’s inability to grow efficiently due to a prolonged lack of oxygen, which may have inhibited its growth. Since growth inhibition limits urease activity, this could explain the observed cementation values and the difference between the top and bottom of the columns. The upper half of the column showed superior cementation, indicating that bacteria could precipitate calcium carbonate and form cementation between sand particles. The combination of B2 and B11 precipitated the highest amount of calcium carbonate among the other samples at the top and bottom half of the column. This result underscores the importance of temperature and bacterial synergies in sand cementation. Notably, the size variance of these bacteria may contribute to this superior cementation, enabling them to infiltrate pores of different sizes and effectively reduce permeability, as suggested by (Bu et al. 2022; Baek et al. 2023; Zhao et al. 2024). Nutrient sharing and cross-feeding interactions among bacterial species may enhance overall metabolic efficiency and substrate utilization. Bacteria can release extracellular enzymes and metabolites that benefit neighboring species, creating a cooperative network within the microbial community. For example, one species may produce urease enzymes that hydrolyze urea into carbonate ions and ammonia, which can be utilized by other bacterial species as nitrogen or carbon sources (Graddy et al. 2021; Li et al. 2023a). This mutualistic interaction promotes metabolic synergy and facilitates more efficient sand cementation and MICP processes.

The findings have significant implications for MICP applications in soil stabilization and erosion control. Enhanced sand cementation and reduced permeability offer promising solutions for stabilizing soil structures, mitigating erosion, and improving ground stability. MICP processes utilizing synergistic bacterial consortia have the potential to provide cost-effective and environmentally friendly alternatives to traditional soil stabilization methods, with applications in construction, geotechnical engineering, and environmental remediation.

**Table 5:** Calcium carbonate precipitation between treatment groups. Groups with the same letter are not significantly different

Sample type	Mean ± SD
B2 R- Top	3.35 ± 1.11c
B2 R-Bottom	1.82 ± 0.14c
B2 30℃ - Top	7.87 ± 0.72ab
B2 30℃-Bottom	3.36 ± 0.10c
B11 R-Top	1.87 ± 0.25c
B11 R-Bottom	1.85 ± 0.06c
B11 30℃-Top	7.39 ± 0.47b
B11 30℃-Bottom	3.36 ± 0.16c
B2&B11 R-Top	3.52 ± 0.69c
B2&B11 R-Bottom	2.37 ± 0.16c
B2&B11 30℃-Top	9.88 ± 0.72a
B2&B11 30℃-Bottom	6.77 ± 0.84b

SD = Standard Deviation

## Conclusion

In conclusion, this study provides valuable insights into the potential of ureolytic bacteria for MICP and its applications in soil stabilization, erosion prevention, and crack repair. The research identifies two *Bacillus Subtilis HMZC1* and *Comamonas fluminis HMZC* bacterial strains with superior urea degradation abilities at an ideal pH of 7 and a temperature of 30 °C. Additionally, the study demonstrated that combining these bacteria caused enhanced urea degradation and calcium carbonate precipitation, highlighting the importance of bacterial synergy in MICP. Our experiments’ quantitative results strongly support these bacterial strains’ efficacy in achieving desirable outcomes. Specifically, the combination of bacterial strains resulted in enhanced urea degradation, reduced permeability, and increased calcium carbonate precipitation. The calcium carbonate content in the cemented sand was significantly higher for the combined bacterial strains at 30 °C compared to individual strains and lower temperatures. Additionally, permeability tests revealed that the combined bacterial strains at 30 °C (1.53 × 10^− 6^ m/s) exhibited the most significant reduction in permeability, indicating superior soil cementation.

Future research directions should focus on exploring the effects of different carrier materials and testing bacterial synergies in more diverse environmental conditions such as clayey, dry, and marine environments. By investigating these factors, we can further optimize MICP techniques for practical applications in engineering projects such as mitigating soil erosion, slope stabilization, and environmental remediation (heavy metal remediation or recovery).

The environmental benefits of MICP as an environmentally friendly technology cannot be overstated. By reducing the need for energy-intensive cement production and lowering carbon emissions, MICP offers a sustainable solution for soil improvement and bioremediation practices. The findings of this study contribute to advancing our understanding of MICP and its potential applications, emphasizing the importance of this innovative technique in addressing contemporary environmental challenges.

In summary, this study underscores the significant contributions of MICP to the field of soil improvement and bioremediation. By harnessing the power of microbial processes, we can create more sustainable and environmentally friendly solutions for engineering projects and environmental conservation efforts.

## Data Availability

The datasets generated during and/or analysed during the current study are available from the corresponding author on reasonable request.
