# Correction to: Synergistic biocementation: harnessing *Comamonas* and *Bacillus* ureolytic bacteria for enhanced sand stabilization

**DOI:** 10.1007/s11274-024-04067-y

**Published:** 2024-07-04

**Authors:** Adharsh Rajasekar, Cailin Zhao, Suowei Wu, Raphinos Tackmore Murava, Stephen Wilkinson

**Affiliations:** 1 Jiangsu Key Laboratory of Atmospheric Environment Monitoring and Pollution Control (AEMPC), Collaborative Innovation Center of Atmospheric Environment and Equipment Technology (CIC-AEET), Nanjing University of Information Science &Technology, Nanjing, 210044 China; 2 School of Geography and Environmental Sciences, University of Reading, Reading, RG6 6AH UK; 3 Faculty of Engineering and Information Sciences, University of Wollongong in Dubai, Dubai, UAE

**Correction to: World Journal of Microbiology and Biotechnology (2024) 40:229**

10.1007/s11274-024-04038-3

In the original publication of the article, the last line under subsection 16s rRNA sequencing, in the materials and methods section has been published incorrectly as:

“The bacterial strains chosen in this study were B2 and B11, and their accession numbers are OQ826692 (similarity index 86.48% and 1229 base pairs) and OQ826707 (similarity index 98.39% and 1198 base pairs). B2 = Bacillus Subtilis HMZC1; B11 = Comamonas fluminis HMZC.”

The correct sentence is:

The bacterial strains chosen in this study were B2 and B11, and their accession numbers are OQ826692 (similarity index 86.48% and 1229 base pairs) and OQ826684 (similarity index 99.93% and 1417 base pairs). B2 = Bacillus Subtilis HMZC1; B11 = Comamonas sp. HMZC.

